# Supplementary material for: SARS-CoV-2 (COVID-19) by the numbers
Source: eLife. 2020 Apr 2;9:e57309. doi: 10.7554/eLife.57309 (PMC7224694; doi:10.7554/eLife.57309)
Supplement: Supplementary file 1. — This is a larger version of Figure 1. [file elife-57309-supp1.pdf]

SARS-CoV-2 (COVID-19) by the numbers

Yinon M. Bar-On[1], Avi Flamholz[2], Rob Phillips[3,4], and Ron Milo[1*]
[1]Weizmann Institute of Science, Rehovot 7610001, Israel [2]University of California, Berkeley, CA 94720, USA
[3]California Institute of Technology, Pasadena, CA 91125, USA [4]Chan Zuckerberg Biohub, San Francisco, CA 94158, USA
[*]Corresponding author: ron.milo@weizmann.ac.il.
Comments are welcome; this article is being updated on an ongoing basis at: https://bit.ly/2WOeN64

eLife
published in eLife, March 31st, 2020
https://elifesciences.org/articles/57309

Size & Content

Diameter: ≈100 nm
Volume: ~10^6 nm^3 = 10^{-3} fL
Mass: ~10^3 MDa ≈ 1 fg

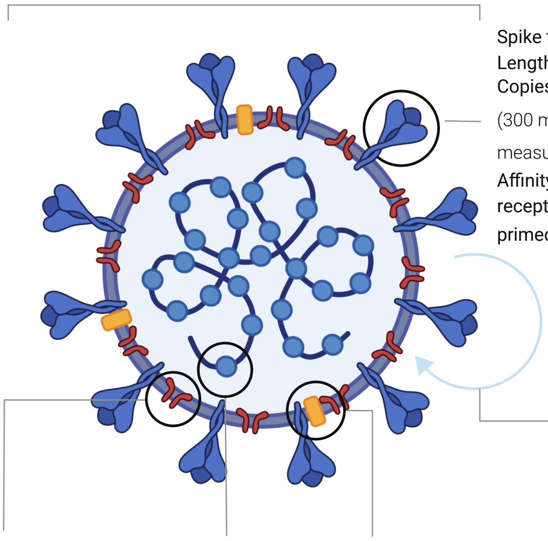

Spike trimer
Length: ≈10 nm
Copies per virion: ≈100
(300 monomers, measured for SARS-CoV-1)
Affinity to ACE2 receptor K_d: ≈1-40 nM
primed by TMPRSS2

Membrane protein
≈2000 copies
(measured for SARS-CoV-1)

Nucleoprotein
≈1000 copies
(measured for SARS-CoV-1)

Envelope protein
≈20 copies
(100 monomers, measured for TGEV coronavirus)

Genome

Nucleotide identity to SARS-CoV-2

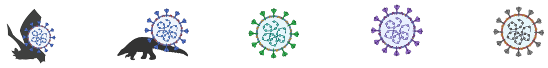

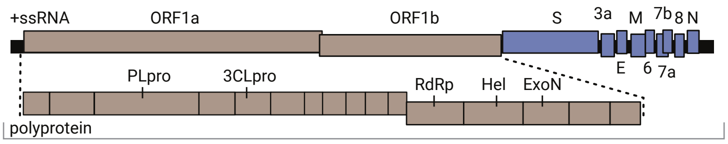

Length: ≈30kb; β-coronavirus with 10-14 ORFs (24-27 proteins)

Evolution rate: ~10^{-3} nt^{-1} yr^{-1} (measured for SARS-CoV-1)
Mutation rate: ~10^{-6} nt^{-1} $cycle^{-1}$ (measured for MHV coronavirus)

Replication Timescales

in tissue-culture
Virion entry into cell: ~10 min (measured for SARS-CoV-1)
Eclipse period: ~10 hrs (time to make intracellular virions)
Burst size: ~10^3 virions (measured for MHV coronavirus)

Host Cells

(tentative list; number of cells per person)
Type I & II pneumocytes (~10^{11} cells)
Alveolar macrophage (~10^{10} cells)
Mucous cell in nasal cavity (~10^9 cells)
Host cell volume: ~10^3 μm^3 = 10^3 fL

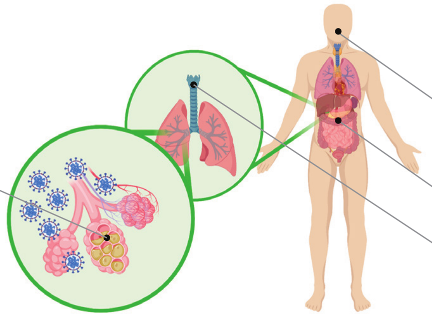

virions not to scale

Concentration

maximal observed values following diagnosis
(Woelfel et al. 2020; Kim et al. 2020; Pan et al. 2020)
Nasopharynx: 10^6-10^9 RNAs/swab
Throat: 10^4-10^8 RNAs/swab
Stool: 10^4-10^8 RNAs/g
Sputum: 10^6-10^{11} RNAs/mL
RNA counts can markedly overestimate infectious virions

Antibody Response - Seroconversion

Antibodies appear in blood after: ≈10-20 days
Maintenance of antibody response:
≈2-3 years (measured for SARS-CoV-1)

Virus Environmental Stability

Relevance to personal safety unclear

	half-life	time to decay 1000-fold
Aerosols:	≈1 hr	≈4-24 hr
Surfaces: e.g. plastic, cardboard and metals	≈1-7 hr	≈4-96 hr
	(van Doremalen et al. 2020)	

Based on quantifying infectious virions. Tested at 21-23Â°C and 40-65% relative humidity. Numbers will vary between conditions and surface types (Otter et al. 2016).
Viral RNA observed on surfaces even after a few weeks (Moriarty et al. 2020).

"Characteristic" Infection Progression in a Single Patient

Basic reproductive number R_0: typically 2-4
Varies further across space and time (Li et al. 2020; Park et al. 2020)
(number of new cases directly generated from a single case)

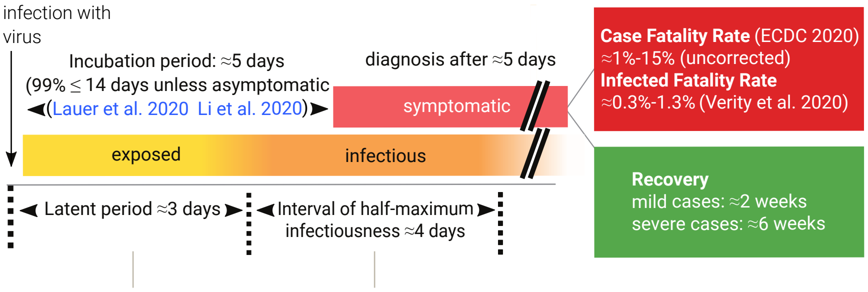

Inter-individual variability is substantial and not well characterized. The estimates are parameter fits for population median in China and do not describe this variability (Li et al. 2020; He et al. 2020).

Note the difference in notation between the symbol ≈, which indicates "approximately" and connotes accuracy to within a factor 2, and the symbol ~, which indicates "order of magnitude" or accuracy to within a factor of 10.
